# A user-friendly database of coastal flooding in the United Kingdom from 1915–2014

**DOI:** 10.1038/sdata.2015.21

**Published:** 2015-05-12

**Authors:** Ivan D. Haigh, Matthew P. Wadey, Shari L. Gallop, Heiko Loehr, Robert J. Nicholls, Kevin Horsburgh, Jennifer M. Brown, Elizabeth Bradshaw

**Affiliations:** 1 Ocean and Earth Science, National Oceanography Centre, University of Southampton, European Way, Southampton SO14 3ZH, UK; 2 School of Civil, Environmental and Mining Engineering and the UWA Oceans Institute, The University of Western Australia, 35 Stirling Highway, Crawley, WA 6009, Australia; 3 Faculty of Engineering and the Environment, University of Southampton, Southampton SO17 1BJ, UK; 4 National Oceanography Centre, Joseph Proudman Building, 6 Brownlow Street, Liverpool L3 5DA, UK; 5 British Oceanographic Data Centre, Joseph Proudman Building, 6 Brownlow Street, Liverpool L3 5DA, UK

**Keywords:** Environmental sciences, Environmental social sciences, Physical oceanography, Atmospheric dynamics

## Abstract

Coastal flooding caused by extreme sea levels can be devastating, with long-lasting and diverse consequences. Historically, the UK has suffered major flooding events, and at present 2.5 million properties and £150 billion of assets are potentially exposed to coastal flooding. However, no formal system is in place to catalogue which storms and high sea level events progress to coastal flooding. Furthermore, information on the extent of flooding and associated damages is not systematically documented nationwide. Here we present a database and online tool called ‘SurgeWatch’, which provides a systematic UK-wide record of high sea level and coastal flood events over the last 100 years (1915-2014). Using records from the National Tide Gauge Network, with a dataset of exceedance probabilities and meteorological fields, SurgeWatch captures information of 96 storms during this period, the highest sea levels they produced, and the occurrence and severity of coastal flooding. The data are presented to be easily assessable and understandable to a range of users including, scientists, coastal engineers, managers and planners and concerned citizens.

## Background & Summary

Flooding of low‐lying, densely populated, and developed coasts can be devastating, with long lasting social, economic, and environmental consequences^1^. These include: loss of life (sometimes in the tens of thousands), both directly and also indirectly (such as due to waterborne diseases or stress-related illnesses); billions of pounds worth of damage to infrastructure; and drastic changes to coastal landforms. Globally, several significant events have occurred in the past decade, including: Hurricane Katrina in New Orleans in 2005^2^; Cyclone Xynthia on the French Atlantic coast in 2010^3,4^; Hurricane Sandy and the New York area in 2012^5–7^; and Typhoon Haiyan in the Philippines in 2013^8^. These events dramatically emphasized the high vulnerability of many coasts around the world to extreme sea levels. Improved technology and experience has provided many tools to mitigate flooding and adapt to the risks. However, as mean sea level continues to rise due to climate change^9,10^, and as coastal populations rapidly increase^11^, it is important that we identify which historic storm events resulted in coastal flooding, where it occurred, and the extent and severity of the impacts.

The UK has a long history of severe coastal flooding. In 1607, it is estimated that up to 2,000 people drowned on low-lying coastlines around the Bristol Channel^12^. This is the greatest loss of life from any sudden-onset natural catastrophe in the UK during the last 500 years^13^. During the ‘Great Storm’ of 1703, the Bristol Channel was again impacted, whilst on the south coast the lowermost street of houses in Brighton was ‘washed away’^14,15^. On 10 January 1928, a storm surge combined with high river flows and caused coastal flooding in central London, drowning 14 people. More recently, the issue of coastal flooding was brought to the forefront by the ‘Big Flood’ of 31 January–1 February 1953, during which 307 people were killed in southeast England and 24,000 people fled their homes^16–18^, and almost 2,000 lives were lost in the Netherlands and Belgium^19^. These events led to widespread agreement on the necessity for a coordinated response to understand the risk of coastal flooding, and to provide protection against such events^20^. The 1953 event in particular was the driving force for constructing the Thames Storm Surge Barrier in London and led to the establishment of the UK Coastal Monitoring and Forecasting (UKCMF) Service^21^. Without the Thames Barrier and associated defences, together with the forecasting and warning service, London’s continued existence as a major world city and financial capital would be precarious^22^. The widespread disruption that can be caused by coastal flooding was again demonstrated dramatically during the northern hemisphere winter of 2013–14, when the UK experienced a series of severe storms^23^ and coastal floods^24^, which repeatedly affected large areas of the coast.

However, there is no formal, national framework in the UK to record flood severity and consequences and thus benefit an understanding of coastal flooding mechanisms and consequences. While the UKCMF produces forecasts of storm surge events four times daily^25^, and continuously monitors sea levels across the National Tide Gauge Network; no nationwide system is currently in place to: (1) record whether high waters progress to coastal flooding; and (2) systematically document information on the extent of coastal floods and associated consequences. Interested parties (e.g., the Environment Agency (EA), local authorities, and coastal groups) often report on events, but detail is usually limited, and the process is unsystematic. Without a systematic record of flood events, assessment of coastal flooding around the UK coast is limited.

As a first step in creating a systematic record of coastal flooding events, we present here a database and online tool called ‘SurgeWatch’. This UK-wide record of coastal flood events covers the last 100 years (1915-2014), and contains 96 storm events that generated sea levels greater than or equal to the 1 in 5 year return level. For each event, the database contains information about: (1) the storm that generated that event; (2) the sea levels recorded around the UK during the event; and (3) the occurrence and severity of coastal flooding as a consequence of the event. The results are easily accessible and understandable to a wide range of interested parties.

## Methods

**Figure 1:**
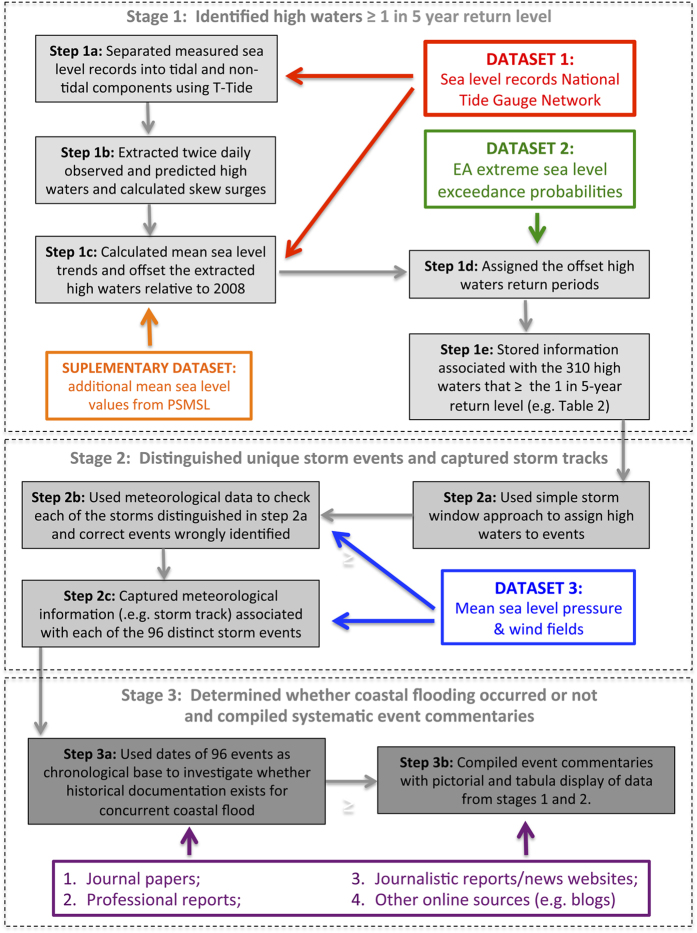
Stages of analysis and data sources. Schematic overview of the multi-staged procedure used to compile the database with data sources.

The database utilizes data from three main sources and involves three main stages of analysis, as explained below and illustrated in Fig. 1.

### Data sources

**Figure 2:**
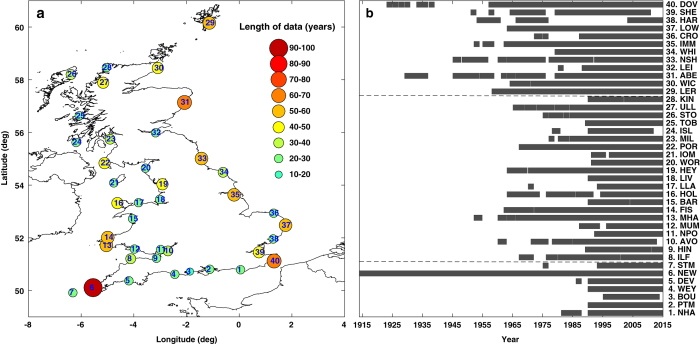
Study sites and record lengths. (**a**) Location of tide gauge sites; and (**b**) duration of sea level records.

The first and primary dataset used is records from the UK National Tide Gauge Network, available from the British Oceanographic Data Centre (BODC) archive (Data Citation 1). We used these records to identify high sea level events that had the potential to cause coastal flooding. This network consists of 43 operational tide gauges, and was set up as a result of the severe flooding in 1953. It is owned by the EA and maintained by the National Oceanography Centre (NOC) Tide Gauge Inspectorate. We used data from 40 of the network’s tide gauges (Fig. 2); two sites in Northern Ireland (Bangor and Portrush), and Jersey in the Channel Islands were omitted. This is because the sea level exceedance probabilities (see description of second data type below) used to assign return periods to high waters, are currently available for only England, Scotland and Wales. The longest record is at Newlyn Cornwall, which started in 1915, and the shortest is at Bournemouth (Dorset), which started in 1996 (Fig. 2; Table 1). Newlyn has been maintained as the principal UK tide gauge since 1915 and is recognised as one of the best quality sea level records in the world^26^. The mean data length for all considered gauges is 38 years. At the time of analysis, quality-controlled records were available until the end of 2014. The data frequency prior to 1993 was hourly and from January 1993 onwards increased to 15-minute resolution.

The second type of data is sea level exceedance probabilities, estimated recently in a national study^27,28^ commissioned by the EA. Exceedance probabilities, often called return periods/levels, convey information about the likelihood of rare event such as floods. For example, a 1 in 50 year return level is where there is a 1 in 50 chance of that level being exceeded in a year. We used these return levels to define a threshold for selecting high waters at each site, that were likely to have resulted in coastal flooding. In the EA study, a method, called the Skew Surge Joint Probability Method (SSJPM), was developed and used to estimate sea level exceedance probabilities at the 40 national tide gauge sites on the English, Scottish and Welsh coasts (and five additional sites where long records were available). A multi-decadal hydrodynamic model hindcast was used to interpolate these estimates around the coastlines at 12 km resolution. We extracted (using the information listed in Table 4.1 of McMillian *et al.*^27^) the return levels for 16 return periods (from 1 in 1 to 1 in 10,000 years), for each of the 40 sites. By interpolating these 16 return periods, at each site, we were able to estimate the return period of each extracted high water.

The third type of data is a global meteorological dataset of mean sea level pressure and near-surface wind fields from the 20th Century Reanalysis, Version 2^29^ (Data Citation 2). We used this data to track storms associated with the high waters that exceeded our chosen threshold (a 1 in 5 year return level). These data are available at a spatial resolution of 2° every 6 h from 1871–2012. 2013 and 2014 are not covered by the 20th Century Reanalysis, so we used a supplementary and similar dataset from the US National Center for Environmental Predictions/National Center for Atmospheric Research’s (NCEP/NCAR) Reanalysis, Version 2^30^ (Data Citation 3). These fields are also available every 6 h (since 1948) but have a horizontal resolution of 2.5°. For consistency, we spatially interpolated the data onto the 2° 20th Century Reanalysis grid. We used the data between latitudes 30°N and 85°N and longitudes 75°W and 20°E; the area where extra-tropical storms that track towards and influence the UK are generated.

### Stage 1: Deriving the high water dataset

The first stage to create the database was to establish when high waters (that were recorded from the available records) reached or exceeded a threshold (for this we used the 1 in 5 year return level, for reasons explained below), at each of the 40 tide gauge sites. This identified events that had the potential to cause coastal flooding.

First, measured sea levels at each of the 40 tide gauge sites were separated into tidal and non-tidal components^31^ so that the relative contribution of tide and surge could later be identified. The tidal component is the regular rise and fall of the sea caused by the astronomical forces of the Earth, Moon and Sun. The non-tidal residual component remains once the astronomical tidal component has been removed. This primarily contains the meteorological contribution termed the surge, but may also contain harmonic prediction errors or timing errors, and non-linear interactions^32^. It is for this reason that we estimate skew surge^29^, rather than the traditionally-used, non-tidal residual at high water. A skew surge is the difference between the maximum observed level and the maximum predicted tidal level regardless of their timing during the tidal cycle. There is one skew surge value per tidal cycle. The advantage of using skew surge is that it is an integrated and unambiguous measure of the storm surge. The tidal component was estimated using the freely available Matlab T-Tide harmonic analysis software^33^ (http://www.eos.ubc.ca/~rich/#T_Tide). A separate tidal analysis was undertaken for each calendar year with the standard set of 67 tidal constituents. For years with less than 6 months of data coverage, the tide was predicted using harmonic constituents estimated for the nearest year with sufficient data.

Second, we extracted all twice-daily, measured and predicted high water levels at each site, as this is the parameter most relevant to flooding. To do this we used a two-staged turning point approach (described in the Technical Validation section). We then calculated skew surges from the measured and predicted high waters.

Third, we offset the extracted high waters by the rate of mean sea level rise observed at each site. This was in order to directly compare the joint probability of the skew surge and astronomical tide (i.e., extremity) of the high water events throughout the record, independently of mean sea level change. This is because the EA return periods are relative to a baseline level, which corresponds to the average sea level for the year 2008^27,28^. At locations that have undergone a rise in mean sea level over the duration of the record, sea levels before 2008 would have a higher return period, and lower return period thereafter^24^. For example, the 5th largest high water in the Newlyn record occurred on 29 January 1948. When this is offset by mean sea level rise (mean sea level was 10 cm lower in 1948 compared to 2008), this high water actually has the largest return period at that site^24^. At each site, we calculated time series of annual mean sea levels, using the high-frequency records from the BODC, supplemented with additional annual mean values obtained from the Permanent Service for Mean Sea Level’s (PSMSL) archive (Data Citation 4). The PSMSL records are longer at certain sites, compared with the high frequency data available from the BODC archive, and for this reason we make use of this additional dataset where available. We estimated trends in mean sea level using linear regression following the method used by Woodworth *et al.*^34^ and Haigh *et al.*^35^ (rates are listed in Table 1). For sites where the data length was too short (<20 years) to accurately estimate trends^35^, we interpolated the trend values from the two surrounding sites. All estimates were checked against results from previous studies of mean sea level changes around the UK^34,35^, and there is good agreement.

Fourth, we linearly interpolated the EA exceedance probabilities and then estimated the return period of every high water, after offsetting for mean sea level, so that we could directly compare events throughout the record.

Fifth, we stored information associated with the measured high waters that were equal to or greater than the offset 1 in 5-year return level threshold, at each of the 40 sites. We chose this threshold, because: (1) tides are large every 4.4 years due to the lunar perigee cycle^36^ and we wanted to ensure events arose as consequence of a storm surge and not just a large tide; and (2) it gave us a manageable number of 96 events in stage 2 (for example, selecting the 1 in 1 year threshold would have given more than 350 distinct events and a large proportion of these are unlikely to have caused coastal flooding). For each offset high water that was equal to or greater than the 1 in 5-year return level threshold, we recorded the: (1) date-time of the measured high water; (2) offset return period; (3) measured high water level; (4) predicted high water level; (5) skew surge; and (6) site number (Table 1). Across the 40 sites (for the period 1915 to 2014), 310 high waters reached or exceeded the 1 in 5-year threshold (the top 20 high waters are listed in Table 2, sorted by decreasing the return period). In addition, we also stored information about the top 20 skew surges at each site. This Supplementary Dataset can be used to access storms that generated large skew surges, but which did not lead to coastal flooding because they occurred, for example, on neap tides.

### Stage 2: Individual storm events

The second stage was to distinguish distinct, extra-tropical storms that produced the 310 high waters that were identified in stage 1, and then to capture the meteorological information about those storms.

To distinguish storms and then assign each of the 310 high waters to one of these, involved a two-stepped procedure. First, we used a simple ‘storm window’ approach. We found that the effect of most storms that cause high sea levels in the UK typically last up to about 3.5 days. We started with the high water of highest return period, and found all of the other high waters that occurred within a window of 1 day and 18 h before or after that high water (i.e., 3.5 days). We then assigned to these the event number 1 (see Table 2). We set all high waters associated with event 1 aside and moved on to the high water with the next highest return period, and so on. This procedure identified 96 distinct events.

**Figure 3:**
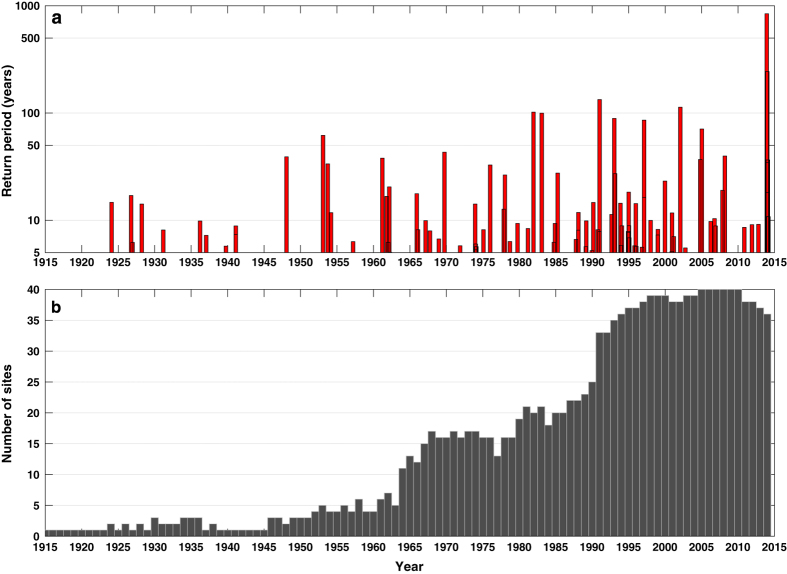
Water level events and data availability. (**a**) Return period of the highest water levels in each of the 96 storm events; and (**b**) the number of sites per annum for which sea level data is available across the 40 sites.

Second, we used the meteorological data to determine if the our above-described procedure had correctly linked high waters to distinct storms. To do this we created an interactive interface in Matlab that displayed the 6-hourly progression of mean sea level pressure and wind vectors over the North Atlantic Ocean and Northern Europe around the time of maximum water level. On all but two occasions, our simple procedure correctly identified distinct storms. However, on 9–10 February 1997, the procedure identified one event, whereas, examination of the meteorological conditions showed that there were two distinct storms that crossed the UK in this period in close succession. Hence, we separated the high waters into two distinct events and altered the event numbers accordingly. In contrast, on 11–13 November 1997, our simple procedure identified two events, whereas there was actually only one event, associated with a particularly slow moving storm. Hence, we merged the high waters into one event, and altered the event numbers accordingly. Using this two-stage procedure, we were able to verify that the 310 high waters identified in stage 1, resulted from 96 distinct storms. For most storms, the 1 in 5 year threshold was reached or exceeded at more than one site, and in some cases two high waters exceeded the threshold during the same storm. The time and maximum return period for each of the 96 events is shown in Fig. 3a.

**Figure 4:**
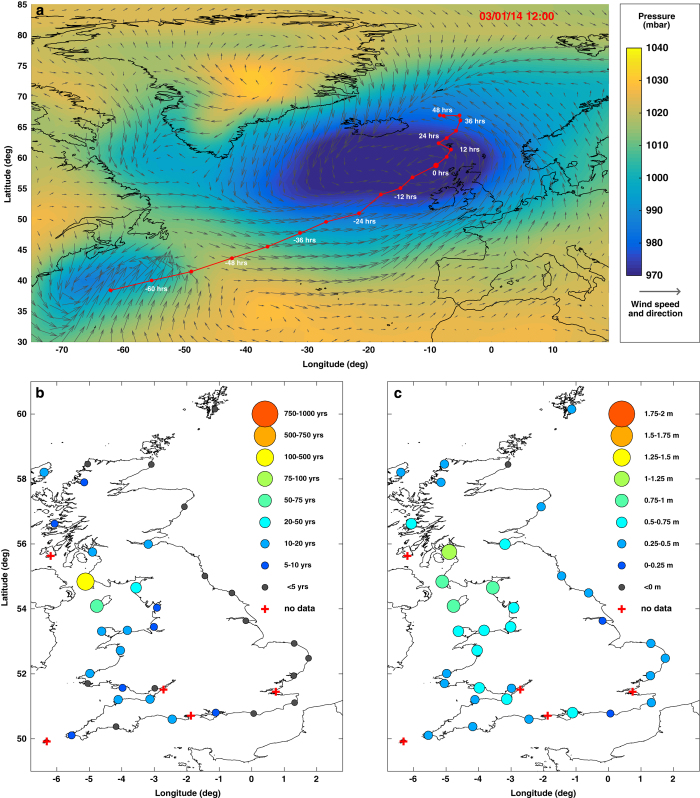
Example event. (**a**) Meteorological conditions at time of maximum water level at Portpatrick and complete storm track; (**b**) water level return period; and (**c**) skew surge levels; for the second largest event in the data record (3rd January 2014; event 2).

Third, we digitized (using our interactive Matlab interface) the track of each of the 96 storms, from when the low-pressure systems developed, until they dissipated or moved beyond latitude 20°E. Different disciplines capture storm tracks in different ways. Because our focus is upon storm surges generated by the low pressure and the strong winds associated with storms, we captured the storm tracks by selecting the grid point of lowest atmospheric pressure at each 6-hour time step. From the start to the end of the storm, we recorded the 6-hourly: (1) time; (2) latitude; and (3) longitude of the minimum pressure cell; and (4) the minimum mean sea level pressure. For example, the storm track of the second largest event in the database is shown in Fig. 4a.

### Stage 3: Coastal flooding

In the third and final stage, we used the dates of the 96 events as a chronological base from which to investigate whether historical documentation exists for a concurrent coastal flood; using a similar approach to that undertaken for the Solent, southern England by Ruocco *et al.*^37^. For each event, we searched a variety of sources for evidence of coastal flooding, including: (1) journal papers; (2) publically available reports and newsletters by interested professional parties such as the EA, Meteorological Office, local councils and coastal groups; (3) journalistic reports/news websites; and (4) other online sources (e.g., blogs, social media). In combination, these helped to establish whether coastal flooding occurred or not during the identified high sea level events. Depending on completeness of the information, we also estimated the extent of flooding and associated damages. Zong and Tooley^38^ and Stevens *et al.*^39^ previously complied lists of floods using similar sources, and we greatly benefitted from these studies.

We also compiled a short but systematic commentary for each event. These contain a concise narrative of the meteorological and sea level conditions experienced during the event, and a succinct description of the evidence available in support of coastal flooding, with a brief account of the recorded consequences to people and property. In addition, these contain a graphical representation of the storm track, mean sea level, pressure, and wind fields at the time of maximum high water (e.g., Fig. 4a). They also include figures of the return period and skew surge magnitudes at sites around the UK (e.g., Fig. 4b,c), and a table of the date and time, offset return period, water level, predicted tide, and skew surge for each site where the 1 in 5 year threshold was reached or exceeded (e.g., Table 3) for each event.

## Data Records

The database presented here (v1.0) is available to the public through an unrestricted repository at the BODC portal (Data Citation 5), and is formatted according to their international standards. This includes data available at the time of publication (up to the end of 2014). There are two files that contain the meteorological and sea level data for each of the 96 events. A third file contains the list of the top 20 largest skew surges at each site. These CSV files are self-describing and include extensive metadata. In the file containing the sea level and skew surge data, the tide gauge sites are numbered 1 to 40 (see Fig. 2). A fourth accompanying CSV file lists, for reference, the site name and location (longitude and latitude). There are also 96 separate PDF files containing the event commentaries.

The database is also freely available at the accompanying SurgeWatch website (http://www.surgewatch.org), with interactive graphical presentations, a glossary of relevant terms, educational videos and news articles and with any subsequent database updates. The database is designed to be updated annually (at the end of each subsequent storm surge season) to include any additional events that reach or exceed the 1 in 5 year return level during the latest year.

## Technical Validation

The database presented here has been created using datasets that are all freely available and easily accessible, and have undergone rigorous quality control and validation prior to being used in SurgeWatch. The primary dataset we use are records from the UK National Tide Gauge Network, which underpins the UKCMF service and is therefore maintained to a high standard. The NOC Tide Gauge Inspectorate regularly examines and levels (to ensure consistent reference to vertical benchmarks) each tide gauge, and responds rapidly to mechanical problems, ensuring limited data outage. The BODC is responsible for the remote monitoring, retrieval, quality-control and archiving of data. They carry out daily remote checks on the performance of the gauges. Data are downloaded weekly, are quality controlled (following international standards of the Intergovernmental Oceanographic Commission^40–43^) and archived centrally to provide long time series of reliable and accurate sea levels for scientific and practical (e.g., navigational) use. The archived data is accompanied by flags, which identify: (1) missing data points (e.g., which may be due to mechanical or software problems); (2) suspect values to be treated with caution; and (3) interpolated values. We excluded all values identified as suspect and undertook extensive secondary checks on all 40 records. While the frequency of the records changed from hourly to 15-minutely after 1993, we deliberately did not interpolate the data prior to 1993 to 15-minute resolution. This was so that the values in the database could be exactly matched back to the original records (and hence can be independently verified).

We used the current national standard guidance in exceedance probabilities to assign return periods to high waters. The EA-commissioned study^27,28^ that produced these, is the latest in a number of related UK investigations from the last six decades (see Batstone *et al.*^28^ and Haigh *et al.*^44^ for a summary) that have contributed significantly to developing and refining appropriate methods for the accurate and spatially coherent estimation of extreme water levels.

A rigorous and reproducible multi-stepped procedure was used to identify storms and high sea level events from the available records, that are likely to have resulted in coastal flooding. We extracted all twice-daily measured and predicted tidal high waters from the sea level records and from these calculated skew surges. Extracting high waters is straightforward at sites with tidal curves that are near sinusoidal, using a turning point approach^45^; but difficult at sites which have distorted tidal curves due to more complex shallow water processes (e.g., sites on the central south coast of England). Hence the method we developed to extract high waters was to first predict the tide at each site from 1915-2014 using only the four main harmonic constituents (K_1_, O_1_, M_2_, S_2_), based upon analysis of the most recent year with greatest data coverage, in most cases 2013. Simple tidal curves predicted with just these four harmonics are near sinusoidal, and hence it was easy to extract all twice-daily high waters, using a turning point approach. We then searched for the maximum measured and predicted tidal levels (calculated as described above using 67 tidal constituents for each calendar year) that occurred three hours either side of these simplistic high levels, at each site. When no data were available, the corresponding measured and predicted high sea levels were assigned ‘not a number’ (NaN) so that time-series of high measured and predicted water levels at each sites were the same length. This allowed us to easily calculate skew surges, by simply subtracting the time series of measured high water from the predicted high water. We undertook extensive visual checks of the extracted measured and predicted high waters at each site. The method was found to be robust at extracting all twice daily values, even at sites with complex tidal curves. The tracks of the 96 storms were manually digitized independently by two people (note, we attempted to automate the process, but due to a number of complexities, digitized each track manually). Any differences were checked and corrected to ensure the storm track was accurately captured.

There are however, a number of unavoidable issues with the database that arise because tide gauge records do not all cover the full 100-year period analyzed (i.e., since the start of the Newlyn record in 1915). It is obvious examining Fig. 3b that we may have missed events before the mid-1980s, and particularly before the mid-1960s, when records were spatially more sparse. We also acknowledge that the ranking (i.e., event number, which is based on sea level return period) of several events is lower than it should be. This is because, while we have data at some sites for these events, tide gauges were not necessarily operational at the time along the stretches of the coastline where the sea levels were likely to have been most extreme. For example, the 31 January–1 February 1953^16–19^ event is ranked 10th, but we know from examining the event in detail, and considering other information sources (Rossiter^16^ in particular), that it should be ranked similar to the 5–6 December 2013, in terms of maximum sea level return period. Only four of the 40 tide gauges were operational at that time: further, one of these failed during the event, just prior to high water, and the other available site (Newlyn) was located away from the areas primarily impacted by the storm surge. Another example is the 14–18 December 1989 event that caused extensive flooding on the south coast^37^. This event is ranked 94th. Based on prior analyses of this event^37^, it should probably be ranked in the top 20 events, but unfortunately none of the tide gauges along the central south coast were operational at that time.

It is for these reasons that we acknowledged in the introduction section that the database presented here is only the first (but never-the-less important) stage in creating a systematic record of high sea level and coastal flooding events for the UK. In the future we plan to build on this strong foundation and enhance the database. There are a number of ways we plan to do this. One is to supplement the database with additional tide-gauge records where available. These tide gauges are operated for example by port authorities (such as at Southampton where a new digitised record has been extended back to 1935)^35^. However, because they do not form part of the National Network, such data can be of lower quality and require extensive and time-consuming quality control measures. That is the main reason we did not include this data at this stage. Another way to overcome the issue of event rankings being lower that they should be, is to supplement the database with heights of high water recorded in older journal papers and reports (or even flood markers on buildings or levels in old photos) for which tide gauge records are not digitally available or have been lost. For example, Rossiter^16^ lists heights of sea levels for the 1953 event at 15 tide gauge sites (only 6 of which are part of the National Tide Gauge Network). This information could be used to supplement the database, where available, increasing the event ranking closer to what they should be. However, again this is a time-consuming task, requiring careful assessment. Because this would be utilizing a single value, rather than hourly or 15-minute times-series, the data would also have to be processed and incorporated (and flagged) in the database in a different way. A particular issue with older events are consistency issues with the vertical datum used at that time, in relation to modern datums. These are reasons we chose to leave this type of addition to a subsequent stage. A final way to ensure that no events are missed, and that all events are ranked appropriately, would be to supplement the records with sea level predictions from a multi-decadal model hindcast (e.g., Haigh *et al.*^46^). This is again something we plan to explore in the future by extending the hindcast for the UK used by McMillian *et al.*^27^ and Batstone *et al.*^28^ back to 1915.

Determining whether coastal flooding did or did not occur during each of the 96 events, and estimating the extent and severity of flooding, also had unique challenges. Many of these challenges were encountered previously by Ruocco *et al.*^37^ where they are discussed in detail. The challenges relate to the disproportional amount of information available for different events and the heterogeneous nature of the reporting. For the database presented here, we undertook a first pass assessment of each of the 96 events to quickly establish whether coastal flooding occurred or not, and where possible, we began an initial assessment of the extent of flooding and associated impact (mainly in relation to people and property). In the future, we plan to examine each event in more detail. We have also developed the accompanying website with the capacity to ‘Crowdsource’ additional information (such as photographs—see usage notes section) and through publicity associated with the site, we hope to uncover additional material (i.e., reports) of which we are not currently aware.

In the database we ranked events by the highest return period recorded for each event. It is important to point out that the extent and severity of coastal flooding is not directly correlated or proportional to the sea level return period, for many obvious reasons. For example, the fact that the damage was so limited during the 5–6 December 2013 event (ranked 1st in our database), compared to the tragedy of 1953, is due to significant government investment in coastal defences, flood forecasting and water level monitoring. Wave conditions are also very important^37^. In the future, we plan to develop another way of ranking the 96 events (and additional future events), based on severity of coastal flooding. Here we plan to build on the concepts developed within Ruocco *et al.*^37^, whereby coastal flooding events were ranked according to 5 severity levels (see Table 2 in Ruocco *et al.*^37^) based on number and types of property and infrastructure impacted. This system would need to be modified to account for the more widespread flooding and associated damages experienced nationally and would also need to account for loss of life (Ruocco *et al.*^37^ did not find any loss of human life recorded in the Solent as a direct result of flooding over the last century, unlike past events on the UK east and west coasts). We recognize that there are significant difficulties ranking by severity given the multiple changes that have occurred over time (e.g., increased investment in coastal defenses and larger populations in the coastal zone^39^) and these would need to be considered.

## Usage Notes

We envisage that our database will be used by academics, coastal engineers, managers, planners, and the wider public, for a range of purposes. We have therefore formatted the data record with the BODC in such a way that users can easily examine: all the events; a single event (search for event number); or all events at a particular site (search for site number). All sea levels in the database are relative to metres Admiralty Chart Datum (ACD), which corresponds to lowest astronomical tide (LAT) at most sites. Some users might wish to covert the levels to Ordnance Datum Newlyn (ODN) which is straightforward using the offsets listed in Table 1.

To facilitate wider and easier accessibility to the database we have built an accompanying website (http://www.surgewatch.org). Using a simple interface, users can browse events by time or location. Selecting ‘by time’ brings up a bar chart showing the dates and relative magnitudes of each of the 96 events, along with a table listing the dates and highest return periods for each event. The columns of the tables can be ordered by date, return period, number of affected sites or site with highest return period. Users can also select a smaller time period on the bar chart (e.g., they might just be interested in the last decade) and the table will update accordingly. Clicking on a row in the table will link through to an event. Each event page contains the referenced event commentary, along with Google Maps showing the return period and skew surge at the sites affected, figures of the storm progression and track, and a table listing the data available for that event. Selecting ‘by location’, brings up a map of the UK showing the 40 tide gauge sites. Users can click on a site, or search for a location and the map will zoom in and show the nearby available tide gauges. Selecting a site will open a new page that gives details of that particular tide gauge record along with a table listing only the events that have impacted that site. Like before, clicking on a row in the table will link through to an event page. There are options on the website to download all the data. Alternatively, users can just download the data for a single event or all of the events that have generated high water levels at a particular site.

The website also contains a glossary that explains, with illustrations, key terms relevant to storms, sea levels and coastal flooding. Each of the columns of the various tables contains information icons, which when pressed given further information and help. There is also a ‘news’ section which will be updated regularly. This contains a variety of material, including: short educational videos describing, for example, what storm surges are; articles on historic events outside of the data record, such as the ‘Great Storm’ of 1703; and interviews with coastal managers or people that have experienced flooding. We also plan to use the website to crowdsource additional information. On every event page, there is a button that users can press to contribute any photos they may have of that event. Photos get moderated before showing up against that event.

## Additional information

**How to cite this article:** Haigh, I.D. *et al.* A user-friendly database of coastal flooding in the United Kingdom from 1915–2014. *Sci. Data* 2:150021 doi: 10.1038/sdata.2015.21 (2015).

## Supplementary Material



## Figures and Tables

**Table 1:** Names and locations of study sites.

**Site Number**	**Site Name**	**Longitude (deg)**	**Latitude (deg)**	**Range**	**Number of Years (Data Range)**	**CD to ODN Conversion (m)**	**MSL Trend (mm/yr)**
1	Newhaven	0.057	50.782	1982–2014	30(33)	−3.52	1.90
2	Portsmouth	−1.111	50.802	1991–2014	24(24)	−2.73	1.60
3	Bournemouth	−1.875	50.714	1996–2013	18(18)	−1.40	1.58
4	Weymouth	−2.448	50.608	1991–2014	24(24)	−0.93	1.60
5	Devonport	−4.185	50.368	1987–2014	25(28)	−3.22	1.60
6	Newlyn	−5.543	50.103	1915–2014	100(100)	−3.05	1.82
7	St Mary’s	−6.317	49.918	1976–2014	22(39)	−2.91	1.90
8	Ilfracombe	−4.112	51.211	1968–2014	40(47)	−4.80	2.00
9	Hinkley	−3.134	51.215	1990–2014	24(25)	−5.90	2.00
10	Avonmouth	−2.713	51.508	1961–2012	40(52)	−6.50	2.00
11	Newport	−2.987	51.550	1993–2014	22(22)	−5.81	2.67
12	Mumbles	−3.975	51.570	1988–2014	24(27)	−5.00	2.70
13	Milford Haven	−5.052	51.707	1953–2014	54(62)	−3.71	2.89
14	Fishguard	−4.984	52.013	1963–2014	51(52)	−2.44	2.53
15	Barmouth	−4.045	52.719	1991–2014	23(24)	−2.44	2.53
16	Holyhead	−4.620	53.314	1964–2014	44(51)	−3.05	2.16
17	Llandudno	−3.825	53.332	1971–2014	22(44)	−3.85	2.41
18	Liverpool	−3.018	53.450	1991–2014	24(24)	−4.93	2.66
19	Heysham	−2.920	54.032	1964–2014	50(51)	−4.90	1.56
20	Workington	−3.567	54.651	1992–2014	23(23)	−4.20	1.91
21	Port Erin	−4.768	54.085	1992–2014	21(23)	−2.75	1.91
22	Portpatrick	−5.120	54.843	1968–2014	47(47)	−1.80	2.26
23	Millport	−4.906	55.750	1978–2014	34(37)	−1.62	1.51
24	Port Ellen	−6.190	55.628	1979–2011	23(33)	−0.19	1.79
25	Tobermory	−6.064	56.623	1990–2014	25(25)	−2.39	2.07
26	Stornoway	−6.389	58.208	1976–2014	36(39)	−2.71	2.11
27	Ullapool	−5.158	57.895	1966–2014	45(49)	−2.75	2.27
28	Kinlochbervie	−5.050	58.457	1991–2014	23(24)	−2.50	2.87
29	Lerwick	−1.140	60.154	1959–2014	56(56)	−1.22	0.15
30	Wick	−3.086	58.441	1965–2014	49(50)	−1.71	1.38
31	Aberdeen	−2.080	57.144	1930–2014	66(85)	−2.25	1.44
32	Leith	−3.182	55.990	1981–2014	27(34)	−2.90	1.83
33	North Shields	−1.440	55.007	1946–2014	60(69)	−2.60	2.36
34	Whitby	−0.615	54.490	1980–2014	35(35)	−3.00	2.35
35	Immingham	−0.188	53.630	1953–2014	56(62)	−3.90	2.35
36	Cromer	1.302	52.934	1973–2014	30(42)	−2.75	2.35
37	Lowestoft	1.750	52.473	1964–2014	51(51)	−1.50	2.35
38	Harwich	1.292	51.948	1954–2014	28(61)	−2.02	2.35
39	Sheerness	0.743	51.446	1952–2010	44(59)	−2.90	1.81
40	Dover	1.323	51.114	1924–2014	65(91)	−3.67	1.90

**Table 2:** The top twenty high waters that exceeded a 1 in 5 year return level.

**Date and Time**	**Largest Return Period (years)**	**Water Level (m)**	**Predicted Tide (m)**	**Skew Surge (m)**	**Site Name**	**Event Number**
06/12/13 00:45	843	8.45	6.81	1.64	Dover	1
05/12/13 19:15	787	9.12	7.50	1.62	Immingham	1
05/12/13 17:15	568	7.32	6.08	1.24	Whitby	1
05/12/13 16:15	405	6.58	5.42	1.08	North Shields	1
03/01/14 12:30	244	5.28	4.38	0.89	Portpatrick	2
05/12/13 22:30	188	4.76	2.79	1.93	Lowestoft	1
05/01/91 15:00	134	5.16	4.06	1.10	Portpatrick	3
01/02/02 14:00	113	5.17	4.41	0.76	Portpatrick	4
13/12/81 21:00	102	15.43	13.35	2.08	Avonmouth	5
01/02/83 01:00	100	11.56	9.82	1.74	Heysham	6
01/02/02 12:45	92	6.86	6.12	0.74	Holyhead	4
11/01/93 12:30	89	3.04	2.49	0.55	Lerwick	7
10/02/97 13:15	86	9.96	9.21	0.75	Workington	8
05/01/91 15:00	81	5.07	3.76	1.31	Millport	3
11/01/93 00:15	78	3.03	2.32	0.70	Lerwick	7
01/02/83 20:00	75	8.68	7.43	1.27	Immingham	6
11/01/05 19:00	71	6.06	4.54	1.52	Tobermory	9
12/01/05 08:30	70	6.28	5.21	1.06	Kinlochbervie	9
01/03/14 12:30	68	6.62	5.82	0.80	Port Erin	2
02/02/83 02:00	65	8.03	6.82	1.21	Dover	6

**Table 3:** High waters that exceeded a 1 in 5 year return level during the second largest event across all studied locations on record (3rd January 2014; Event 2).

**Site Name**	**Date and Time**	**Return Period (Years)**	**Water level (m)**	**Predicted Tide (m)**	**Skew Surge (m)**
Portsmouth	03/01/14 12:30	7	5.49	4.91	0.58
Weymouth	03/01/14 08:00	13	2.94	2.53	0.41
Newlyn	03/01/14 06:00	9	6.32	5.96	0.32
Ilfracombe	03/01/14 07:00	20	10.5	10.02	0.48
Hinkley Point	03/01/14 08:00	13	13.35	12.71	0.65
Mumbles	03/01/14 07:15	9	10.73	10.21	0.5
Fishguard	03/01/14 08:00	17	5.8	5.27	0.49
Barmouth	03/01/14 09:15	18	6.36	5.62	0.74
Holyhead	03/01/14 11:00	20	6.74	6.15	0.54
Llandudno	03/01/14 12:00	17	8.93	8.38	0.52
Liverpool	03/01/14 12:00	8	10.79	10.21	0.57
Heysham	03/01/14 12:15	9	11.18	10.47	0.71
Workington	03/01/14 12:30	46	9.9	9.06	0.84
Port Erin	03/01/14 12:30	68	6.62	5.82	0.79
Portpatrick	03/01/14 12:30	244	5.28	4.38	0.89
Millport	03/01/14 13:15	18	4.8	3.71	1.07
Tobermory	03/01/14 07:15	8	5.73	5.05	0.65
Stornoway	03/01/14 08:15	14	5.89	5.4	0.44
Ullapool	03/01/14 08:00	9	6.25	5.77	0.48
Leith	04/01/14 16:15	10	6.52	5.95	0.51
